# Measles outbreak in an occupational setting: challenges to regain elimination status for Spain, 14 January to 11 April 2026

**DOI:** 10.2807/1560-7917.ES.2026.31.24.2600458

**Published:** 2026-06-18

**Authors:** Esteban Aznar, Aurora Fernández-García, Noemí López-Perea, Josefa Masa-Calles, Nazaret Díaz-Sánchez, María Encarna Sánchez Carratalá, Eva Martín-Aragón Gónzalez, Israel Cremades Bernabeu, Gian Pietro Maletta Talarico, María Beso Delgado, Empar Giner Ferrando, Bernardo R Guzmán Herrador, Pilar Soler Crespo, María José Sierra Moros, Fernando Márquez, Esther Córdoba Deorador, Ana María Humanes Navarro, Ana Boned-Ombuena, María Amparo Pascual del Pobil Ferré

**Affiliations:** 1 Coordinating Centre for Health Alerts and Emergencies, Directorate General of Public Health, Ministry of Health, Madrid, Spain; 2 European Programme for Intervention Epidemiology Training (EPIET), European Centre for Disease Prevention and Control (ECDC), Stockholm, Sweden; 3 National Centre for Microbiology, Instituto de Salud Carlos III, Madrid, Spain; 4 Spanish Consortium for Research in Epidemiology and Public Health (CIBERESP), Instituto de Salud Carlos III, Madrid, Spain; 5 National Centre for Epidemiology, Instituto de Salud Carlos III, Madrid, Spain; 6 Public Health Center, Epidemiology Section, Alicante, Spain; 7 Directorate-General for Public Health, Regional Ministry of Health, Valencia, Spain; 8 CIBER in Infectious Diseases (CIBERINFEC), Madrid, Spain; 9 Directorate-General for Public Health, Regional Ministry of Health, Madrid, Spain; * These authors contributed equally to this work and share last authorship.

**Keywords:** Measles, Outbreak, Elimination, Serology, Occupational setting

## Abstract

In January 2026, 2 days after the World Health Organization (WHO) Regional Verification Commission (RVC) for the European Region declared the re-establishment of endemic measles transmission in Spain, an outbreak linked to an office building, occurred in the city of Alicante, with 34 cases (median age: 44 years; interquartile range (IQR): 43–47) including 23 women and 11 men. Key factors shaping this outbreak underscore challenges and possible ways to regain transmission control, which might have relevance for re-achieving elimination status in Spain.

In January 2026, the annual evaluation of the World Health Organization (WHO) Regional Verification Commission (RVC) for the European Region concluded that the situation of measles had worsened in several countries of the Region, reverting to endemic transmission. This was the case in Spain, which had achieved elimination in 2016 [1,2].

## Outbreak detection

On 27 January 2026 a local hospital in Alicante (Valencian Autonomous Community) reported a laboratory-confirmed measles case to the local public health centre (PHC). The patient resided in Alicante, had no history of travel, and had attended work during the infectious period.

On the same day, the PHC contacted the company where the case worked (company A), and 13 additional individuals were suspected of having measles. On 28 January, the PHC declared an outbreak and initiated its investigation, with the support of the regional public health authorities. In the preceding days (25−27 January), three of six company A employees with fever (body temperature > 38°C) and rash who had sought healthcare, were considered as a possible diagnosis.

On 29 January, an additional laboratory-confirmed case was detected in another company located on adjacent floors in the same building (company B). Both companies operated in open-plan office settings with common workspaces and shared break areas. They had 219 and 199 employees respectively.

## Outbreak description and public health actions

**Figure 1:**
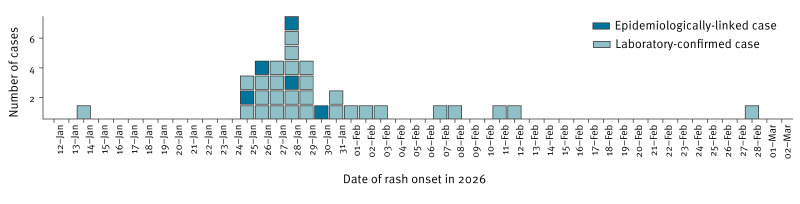
Distribution of measles cases according to their rash onset date and classification, Alicante, Spain, 12 January−2 March 2026^a^ (n = 34 cases)

^a^ The outbreak lasted from 14 January (rash onset of the primary case) to 11 April, but as the graph is based on the dates of cases’ rash onset, it covers a period from 12 January to 2 March.

By the end of the outbreak (11 April), a total of 34 cases had been identified: 29 laboratory-confirmed and five epidemiologically-linked cases, with rash onset dates 14 January–28 February (Figure 1). 

In the context of the outbreak, the standard case definitions from the national surveillance protocol [3] were adapted so that any individual presenting with at least one compatible symptom for measles and an epidemiological link to a confirmed case was considered a suspected (and epidemiologically-linked) case and referred for laboratory tests.

In the laboratory, testing for measles-specific immunoglobulin IgM and IgG in serum and for measles virus RNA in throat swab and urine samples was conducted. According to the national surveillance protocol, laboratory-confirmed cases are defined by at least one of the following criteria: detection of measles-specific IgM antibodies in serum, detection of measles virus RNA by RT-PCR in a clinical sample, isolation of the virus, or significant rise in measles-specific IgG antibodies (seroconversion between acute and convalescent samples). All 29 laboratory-confirmed cases tested positive for measles virus RNA by RT-PCR and 17 of them tested also positive for measles-specific IgM. 

A total of 22 cases, including two epidemiologically-linked cases and 20 laboratory-confirmed cases, showed measles-specific IgG early detection (within the first 7 days after rash onset). 

None of the outbreak cases had travelled abroad during the time within their maximum incubation period of 23 days.

Among 219 employees of company A, 16 cases were identified (attack rate: 7.2%) and among 199 employees of company B, 13 (attack rate: 6.5%).

**Figure 2:**
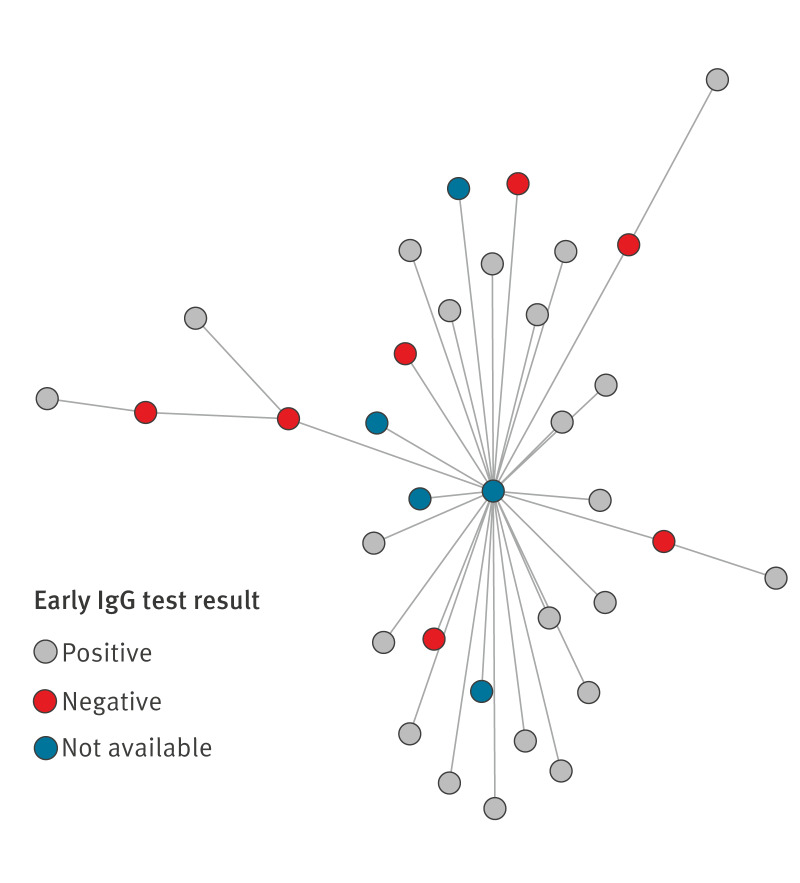
Proposed transmission chains in an outbreak of measles, Alicante, Spain, 14 January−11 April 2026 (n = 34 cases)

The earliest onset of rash occurred on 14 January in the primary laboratory-confirmed case, who worked at Company A. Subsequently 28 additional cases occurred within the two companies; serial interval analysis suggested that all could have been secondary cases, although different transmission chains within the building cannot be excluded. Of the four tertiary cases identified outside the workplace, three were detected among 59 identified social and household contacts, and one was a healthcare worker at a hospital where some cases had been treated. One of these tertiary cases subsequently transmitted the disease to an additional case (Figure 2).

IgG: immunoglobulin G.

Circles represent individual cases and lines indicate probable transmission events. Connections between cases reflect epidemiological links and do not represent temporal or spatial distance.

The median age of cases was 44 years (interquartile range (IQR): 43–47), 23 (67.6%) were women and 11 (32.4%) were men. Measles vaccination status was assessed through the regional registry [4] or, when available, through vaccination records provided by cases. Among the 34 cases, 22 had unknown vaccination status, six were unvaccinated, four had received one vaccine dose, and two were fully vaccinated (Table 1).

**Table 1:** Early IgG detection^a^ among cases of a measles outbreak, according to their vaccination status, Alicante, Spain, 14 January−11 April 2026 (n = 34 cases)

Vaccination status	Number of cases with		
	Negative IgG	Positive IgG	No sample available
Unknown	3	16	3
Unvaccinated	2	2	2
1 dose	2	2	0
2 doses	0	2	0

^a^ Early IgG detection is detection of IgG within the first 7 days after rash onset.

Twenty cases presented with a classical measles clinical picture, including 11 of the 22 cases with early IgG [5]. The remaining 14 cases presented with a modified clinical presentation [5].

Two cases required hospitalisation and recovered. Neither of them had been fully vaccinated or showed early IgG detection.

All tertiary and quaternary cases, who were identified outside the building got measles through contact with secondary and tertiary cases, respectively, who had unknown or partial vaccination status and no evidence of early IgG (Figure 2); early IgG could not be assessed for the primary case. Among contacts identified outside the building, the secondary attack rate was 0% (95% CI: 0.0–9.7) from individuals with detection of early of IgG, compared with 17.4% (95% CI: 5.0–38.8) from individuals with no detection of early IgG (p = 0.02) (Table 2).

**Table 2:** Secondary attack rate among social and household contacts outside of the building where the outbreak occurred, according to the early IgG positivity status of the source cases, Alicante, Spain, 14 January−11 April 2026 (n = 59 contacts)

Source case status	Number of		Secondary attack rate (CI95%)	p value^a^
	Cases arising from the source cases	Identified contacts of the source cases		
Positive early IgG	0	36	0.0% (95% CI: 0.0–9.7%)	0.02
Negative early IgG	4^b^	23	17.4% (95% CI: 5.0–38.8%)	

CI: confidence interval; IgG: immunoglobulin G.

^a^ Fisher's exact test.

^b^ The four cases include three tertiary cases who got measles from secondary source cases with negative early IgG (Figure 2) and one quaternary case who got measles from a tertiary source case with negative early IgG (Figure 2). One tertiary case who was infected via a secondary source case with negative early IgG was not initially identified as a contact of this source case, so was not included in the secondary attack rate estimation (Figure 2).

Samples from the 29 confirmed cases were sent to the National Centre for Microbiology for genomic characterisation. Genotyping was successful in 25 cases. All characterised sequences belonged to the named strain MVs/Ontario.CAN/47.24 (D8) (Dsid: 9171). Sequences were submitted to the WHO Global Measles Nt Sequence Database (MeaNS2; https://whogmrln.org/means2).

To control the outbreak standard measures were implemented including isolation of cases, contact tracing, verification and update of vaccination status among contacts, and post-exposure prophylaxis. In the workplace setting, all employees in the affected building were considered as potential contacts and managed in coordination with occupational health services. The management included assessment of employees’ immune status, targeted vaccination of susceptible individuals, and recommendations to use a mask and telework. Given the magnitude of the outbreak, all healthcare facilities in the region were alerted to enhance the sensitivity of surveillance for outbreak-related cases.

## Suspected origin of the outbreak

On 12 February, regional public health authorities from the Community of Madrid reported a case that could be the source of the outbreak. This adult had had social contact with the primary case in the building on 30 December 2025 and had no recent history of international travel. No definitive source of infection could be identified despite an epidemiological investigation. Compatible symptoms developed on 31 December, and the patient was hospitalised between 4 and 8 January, without measles being suspected.

On 4 February, during a subsequent medical review, serological testing was requested, yielding positive IgM and IgG results on 9 February. On 12 February, a urine RT-PCR test was performed; although the initial result was positive, this could not be confirmed at the national reference laboratory. Hence, while this case was laboratory-confirmed, the measles virus strain affecting the case was not genetically characterised.

## Discussion

The outbreak described in this report has a particular epidemiological relevance for Spain, as it occurred following the country’s recent loss of measles elimination status [6,7]. It took place in an office building with open-plan workspaces, which, together with limited office ventilation during the winter months, likely contributed to extensive transmission from a single primary case [8].

Other elements might have also played a role in the spread of measles virus. Six cases among the 12 with known vaccination status had not been vaccinated, and four had only been vaccinated with one dose, potentially rendering them vulnerable to infection, if they had not prior had the disease. Moreover, the age distribution of the workforce affected by the outbreak covered people susceptible to measles. According to the most recent national seroprevalence study conducted in 2018 [9], the IgG seroprevalence in Spain among those born between 1978 and 1987 was 91.5%, with immunity gaps noted in this group. Indeed, individuals born during these years experienced the transition that occurred when routine measles vaccination was progressively being introduced in the country from the early 1980s, resulting in pockets with heterogeneous immunity profiles depending on their prevalence of past natural infections and vaccination coverage. In this context, maintaining high two-dose vaccination coverage remains the cornerstone of measles control.

While the public health relevance of waning immunity for measles remains debated, people born from the late 1970s to the late 1980s potentially have a higher likelihood of breakthrough infections, possibly reflecting waning immunity in individuals vaccinated decades ago in the absence of natural boosting [9,10]. Nevertheless, during the outbreak, a substantial proportion of cases showed early detection of measles-specific IgG, which may indicate infection in individuals with pre-existing immunity (e.g. reinfection or breakthrough infection). Notably, no onward transmission was observed from cases with early IgG detection, suggesting a lower transmission potential in these individuals.

However, early IgG detection cannot be uniformly interpreted and does not allow differentiation between one- and two-dose vaccination. In this regard, nearly two-thirds of the total cases (i.e. 22/34) had an unknown vaccination status. Among them nine cases were born outside Spain, introducing additional uncertainty in the interpretation of individual protection. Overall, and in the absence of complete vaccination records, cases with missing vaccination status are likely to include a mixture of unvaccinated and partially vaccinated individuals, which may partly explain the observed attack rate in the setting currently described.

Another element that could have contributed to the dissemination of the virus in the current outbreak, was that measles was not initially suspected in several cases in its early stage. This illustrates how the disease may be overlooked in adults, where it is less frequently considered in the differential diagnosis, particularly in elimination settings where low incidence reduces clinical familiarity with measles presentation. Moreover, in elimination settings, many prior vaccinated/infected cases are expected to present with modified measles, characterised by milder or atypical clinical manifestations [5]. These cases pose significant diagnostic challenges, particularly in settings with limited clinical familiarity, potentially delaying recognition and control. While modified measles is generally associated with reduced transmissibility [11], delayed detection may still facilitate ongoing transmission. Hence, ensuring timely identification and investigation of people with any symptoms suggesting measles, appears more critical than considering additional vaccination beyond two doses.

The virus variant involved in this outbreak is the MVs/Ontario.CAN/47.24 named strain belonging to genotype D8, which had been widely circulating in several countries in the Americas in the preceding months [12]. However, the investigation did not identify a definitive link to any imported case.

The limitations related to several cases not being diagnosed at their first contact with healthcare services before the declaration of the outbreak, unknown vaccination status among a proportion of outbreak cases and the inability to establish a definitive epidemiological link to an imported case, highlight further measures that can be taken to prevent future epidemics. These include strengthening surveillance sensitivity. In a context of re-establishment of endemic transmission, a lower threshold for laboratory testing upon clinical suspicion is warranted, as the positive predictive value of clinical diagnosis increases with incidence. Although the current national surveillance protocol already recognises modified measles as a valid clinical criterion in fully vaccinated individuals [3], many individuals in the affected age groups lack documented vaccination records. Extending this consideration to partially vaccinated individuals and those with unknown vaccination status could improve surveillance sensitivity. Systematic assessment of vaccination status and provision of vaccination to individuals with incomplete or unknown immunisation histories should be encouraged. Strengthening and retrospectively completing vaccination registries is also essential, as incomplete records limit the ability to interpret outbreaks and guide targeted interventions, particularly in the context of increasing adult cases.

## Conclusion

As Spain is currently in the process of updating its National Measles and Rubella Elimination Plan, outbreak descriptions such as this one provide important insights to strengthen key areas of action, with the aim of restoring the interruption of endemic measles transmission and consolidating progress towards elimination.

## Data Availability

Individual-level data cannot be shared publicly due to legal and ethical restrictions related to personal data protection in the context of public health surveillance. All relevant aggregated data are included in this published article and its tables. Viral sequences generated in this study were submitted to the WHO Global Measles Nucleotide Sequence Database (MeaNS2). Additional anonymised and aggregated data may be available from the corresponding author upon reasonable request, subject to approval by the competent public health authorities.
